# Effect of rabbit gastrointestinal stasis (RGIS) on the fecal microbiota of pet rabbits (*Oryctolagus cuniculus)*

**DOI:** 10.1371/journal.pone.0318810

**Published:** 2025-02-25

**Authors:** Faith M. Rahic-Seggerman, Cayla Iske, Jennifer Graham, Nicole Furst, Stephan Schmitz-Esser, Micah R. Kohles

**Affiliations:** 1 Department of Animal Science, Iowa State University, Ames, Iowa, United States of America; 2 Omaha’s Henry Doorly Zoo and Aquarium, Omaha, Nebraska, United States of America; 3 Graham Veterinary Consulting, LLC, 119 Sally Lane, Madison, Alabama, United States of America; 4 VCA Abbott Animal Hospital, Worcester, Massachusetts, United States of America; 5 Oxbow Animal Health/Compana Pet Brands, Omaha, Nebraska, United States of America; University of Missouri, UNITED STATES OF AMERICA

## Abstract

Rabbit (*Oryctolagus cuniculus*) gastrointestinal stasis syndrome (RGIS) is defined as reduced motility of any part of the digestive tract that can lead to impaction and death if left untreated. This study aimed to describe the effect of RGIS on the fecal microbiota of client-owned pet rabbits. Fecal samples from healthy rabbits and rabbits displaying RGIS were obtained and the symptomology of the rabbits was recorded along with any medical intervention. The health outcomes were as follows: 1) Healthy rabbits (Healthy, n = 21), and 2) Rabbits that displayed symptoms of RGIS, were treated, and recovered (RGIS, n = 22). The fecal samples were analyzed using 16S rRNA and 18S rRNA gene amplicon sequencing to characterize the bacterial and eukaryotic microbial communities, respectively. In the 16S rRNA amplicon dataset, two bacterial genera were found in higher abundance in rabbits with RGIS: *Clostridium sensu stricto 1* and an unclassified genus in the *Enterobacteriaceae* family. Likewise, five genera were found in higher abundance in healthy rabbits. The yeast *Cyniclomyces guttulatus* dominated the eukaryotic microbiota in all rabbits. RGIS is one of the most common issues in clinical practice. This study is the first to perform detailed characterization of the effects of RGIS on the domestic rabbit’s bacterial and eukaryotic fecal microbiota. The results demonstrate a significant change in the relative abundance of seven bacterial genera associated with RGIS. Future research is necessary to elucidate the potential role of these microorganisms in RGIS. In the long-term, treatments targeting the restoration of the physiological gastrointestinal microbiota should be developed.

## Introduction

Rabbits are hindgut fermenters and as such rely on the microbiota in their comparatively large cecum to ferment plant structural carbohydrates from their herbivorous diet. This fermentation yields volatile fatty acids (VFAs) and other fermentation products that are absorbed by the animal for energy [1]. Both fermentable and non-fermentable fibers are critical for maintaining a healthy gastrointestinal (GI) tract in the rabbit [1–3]. Dietary fiber is crucial for maintaining normal intestinal motility. Fiber stimulates motility either by its conversion by the cecal microbiota to VFAs that promote peristalsis or directly by its physical particle size [2,3]. The cecal microbiota also serves as one of the main sources of amino acids for the rabbit after fecal pellets are expelled and re-ingested in a process known as cecotrophy [1]. Along with procuring energy from food, the GI tract microbiota has a pivotal role in regulating host immunity and influencing GI motility [4]. Many of these interactions can be mediated by microbiota-associated signaling molecules that have the capacity to act on receptors throughout the host [5,6]. More specifically, the signaling molecules can interact with receptors linked to the enteric nervous system, meaning these microbially-derived molecules have the potential to further influence GI motility of the host.

RGIS is defined as reduced motility of any part of the digestive tract that can lead to impaction and death if left untreated [2]. Impaired GI motility can cause ingesta and hair to accumulate, which can lead to a drop in pH. It is speculated from other rabbit pathologies, mainly epizootic rabbit enteropathy (ERE), that potentially pathogenic bacteria such as *Clostridium* spp. or *Escherichia coli* may proliferate in response to the shift in pH [2,7–10]. The proliferation of these microorganisms could be the source of the gas accumulation frequently observed in RGIS. In response to the discomfort, the rabbit will often refuse food, water, or movement which exacerbates the stasis. The etiology of RGIS is multifactorial, but known triggers include stress, dehydration, excess carbohydrates, anorexia, rapid changes in diet, and a lack of long strand dietary fiber [2]. With proper medical intervention, most RGIS cases make a full recovery. If left untreated, severe cases of RGIS can cause death in under 24 hours. RGIS is one of the most common issues in clinical practice, with one retrospective study reporting that over 25% of rabbit patients presented with RGIS [11]. It is currently unknown if the GI microbiota might be associated with RGIS. If dysbiosis of the GI tract microbiota is associated with RGIS, treatments targeting the restoration of the GI microbial populations and environment to normalcy should be implemented. Here, we analyzed the effect of RGIS on the fecal bacterial and eukaryotic microbiota of pet rabbits.

## Materials and methods

### Animal subjects and experimental design

Rabbits (*Oryctolagus cuniculus*) used in this study were client-owned rabbits presenting through the Cummings School of Veterinary Medicine at Tufts University, the Avian and Exotic Animal Clinic of Indianapolis; Griffin Exotics, Kannapolis, NC; Avian and Exotic Animal Hospital of Georgia; Center for Avian and Exotic Medicine New York City. and were divided into two groups: 1) normal healthy rabbits and 2) rabbits presenting with rabbit gastrointestinal stasis syndrome (RGIS). The collection of fecal samples and applicable patient data including owner consent was reviewed and approved by the Cummings School of Veterinary Medicine Clinical Studies Review Committee. Rabbits were classified as having RGIS based on a history of decreased appetite and/or fecal production, decreased activity, and evidence of abdominal pain, including reluctance to move, teeth grinding, hunched body position, and guarding or splinting upon abdominal palpation. A client questionnaire covering diet, husbandry, and medical history along with examination findings including patient body condition score were recorded. Rabbits were excluded from the study if: they had received antibiotic treatment within the past three months, had received other concurrent disease diagnoses, had incomplete husbandry or medical information, or if shipped fecal samples were received at room temperature. Fecal samples from 48 rabbits (29 male, 14 female) were collected over the course of the study. The age of the rabbits ranged from four months to ten years old and it was ensured that all participating rabbits received a similar diet. The control group consisted of clinically healthy rabbits in the group labeled Healthy (n = 21). Rabbits that presented with RGIS, were treated, recovered, and were included in the group RGIS (n = 22).

### Sample collection

Fresh, hard fecal pellets were opportunistically collected from the rabbit’s transport carrier or upon defecation in the exam room or during hospitalization. Fecal samples were collected in a sterile plastic tube and then immediately frozen at –20°C. The samples were shipped on dry ice to Iowa State University (ISU) and stored at -80°C.

### DNA extraction

The samples were thawed at room temperature before one pellet was transferred into a sterile 1.5 mL microtube. Pellets were weighed, and additional pellets were added to reach a weight of approx. 0.2 g. The hard-fecal pellet was ground with a sterile pestle until the pellets were homogenous. 750 µ L of sterile 1x phosphate buffered saline (PBS) was added and vortexed for 30 seconds before transferring into a sterile bead tube. DNA was extracted from the prepared pellet using the standard protocol of the Qiagen DNeasy Powerlyzer Powersoil kit (Qiagen, Germantown, MD). This included utilizing a Fisher Scientific Beadmill 24 for mechanical cell lysis. After all fecal samples were processed, three samples of sterile PCR-grade water (250 µ L) were processed with the same kit. These samples were sequenced alongside the fecal samples to account for possible contamination during the DNA extraction, amplification, or sequencing procedures. The concentration of the isolated DNA was measured using a ND-100 Nanodrop spectrophotometer (NanoDrop Technologies, Dockland, DE). Before sequencing, the DNA concentrations were adjusted to 25 ± 5 ng/ µ L.

### 16S rRNA and 18S rRNA gene amplicon sequencing

16S rRNA and 18S rRNA gene amplicon sequencing was completed using the Illumina MiSeq platform at the Iowa State University DNA facility. A modified version of the 16S/18S Illumina amplicon protocol created by the Earth Microbiome Project was followed [12]. Universal 16S rRNA gene bacterial primers 515F (5′-GTGYCAGCMGCCGCGGTAA-3′) and 806RB (5′-GGACTACNVGGGTWTCTAAT-3′), targeting the V4 variable region, were used. Primers V8f forward (5’-ATAACAGGTCTGTGATGCCCT-‘3) and EukBr reverse (5’-TGATCCTTCTGCAGGTTCACCTAC-‘3) were used to amplify the V8-V9 variable regions of the 18S rRNA gene. The 16S rRNA samples underwent PCR using the Platinum Hot Start PCR Master Mix (2x) (ThermoFisher) and the following conditions: initial denaturation step at 94 °C for 3 min; 45 s at 94 °C; 60 s at 50 °C; 90 s at 72 °C. This was repeated for 35 cycles and finished with a 10 min extension at 72 °C. The 18S rRNA samples also underwent PCR using the Platinum Hot Start PCR Master Mix and the following conditions were used: initial denaturation at 94 °C for 3 min; 45 s at 94 °C; 60 s at 57 °C; 90 s at 72 °C. These steps were repeated for 35 cycles and finished with a 10 min extension at 72 °C. After PCR, the amplicons were purified using the standard protocol of the QIAquick 96 PCR Purification Kit (Qiagen, Hilden, Germany). All described reagents were dispensed using a Mantis robot (Formulamatrix). The barcoded amplicons underwent paired-end 500-cycle sequencing on an Illumina MiSeq platform. Reads were demultiplexed by the facility.

### Sequence analysis

The 16S and 18S rRNA gene amplicon sequences were processed and analyzed separately using the following protocol. Raw sequences were processed using the mothur (v1.48.0) [13] software following a protocol based on the MiSeq Standard Operating Procedure [14]. The “make.contigs” command was used to merge and filter the paired-end reads. Parameters including a maximum homopolymer run of eight bp, a minimum length of 252 bp and 328 bp for the 16S and 18S rRNA datasets respectively, and a cutoff of zero ambiguities were applied for the “screen.seqs” command. Sequences were aligned using the SILVA reference database (v138) [15] and the “align.seqs” command. Chimeric sequences were removed using the “chimera.vsearch” command in combination with the SILVA.gold reference. *De novo* operational taxonomic unit (OTU) clustering at 99% gene similarity was completed and the resulting OTUs were classified using the SILVA reference database (v138). For the 18S rRNA dataset, OTUs classified as vertebrates, plants, or arthropods were removed using the command “remove.lineage”. The resulting OTU data was imported into R for subsequent analysis. The package decontam (v1.20.0) [16] was used to identify and remove OTUs that were prevalent in the PCR-grade water control samples. Microbial community visualization was completed using the Phyloseq (v1.38.0) [17], vegan (v2.5.7) [18], and ggplot2 (v3.4.0) [19] R packages.

### Statistical analysis

For both the 16S and 18S rRNA dataset, OTUs with fewer than 10 reads were removed prior to statistical analysis. One sample from the 18S rRNA dataset with fewer than 3,000 reads after quality control was removed due to insufficient read depth. SAS (Version 9.4, SAS Inst., Cary, NC) was used to analyze a full model and a reduced model. The full model included the fixed effects of Age, Sex, Health Status, and the two and three-way interaction(s) between the effects. The reduced model included the fixed effect of Health Status. The reduced model was found to have significantly lower Bayesian information criterion (BIC) values and was used for all subsequent analysis.

Differences in beta diversity based on the health status of the animal were analyzed using Bray-Curtis distances and visualized using principal coordinate analysis (PCoA) plots. A permutational multivariate analysis of variance (PERMANOVA) and a permutational multivariate analysis of dispersion was completed with the commands “adonis2” and “betadisper” from the vegan package in R [18]. The resulting p-values were adjusted to account for multiple-comparisons using Bonferroni’s correction [20].

To evaluate the effect of the animal’s health status on microbial community structure, the Phyloseq package in R was used to generate alpha diversity measurements for the number of observed species, Chao species richness, Simpson evenness, and Shannon diversity [17]. The least squares means (LSMeans) of all alpha diversity measurements for each sampling group were compared using the MIXED procedure in SAS [21]. The resulting p-values from the pairwise comparisons were adjusted for multiple comparisons using Tukey’s Honest Significant Difference test [22]. Adjusted p-values were considered significant if p < 0.05.

The “tax_glom” command in Phyloseq was used to generate phylum and genus level bar graphs. The relative abundance of the 10 most abundant phyla and 15 most abundant genera were included in the analysis. The GLIMMIX procedure of SAS was used to evaluate the fixed effect of Health Status on the relative abundance of genera and individual OTUs. The MULTTEST procedure within SAS was implemented to control for the false discovery rate, and results were considered significant if q < 0.05. For genera or OTUs where q < 0.05, the Log_2_fold-changes between treatment groups were calculated and plotted using R.

## Results

### 16S rRNA gene amplicon sequencing

After quality control, the average sequencing depth per sample was 129,559 with a standard deviation of 25,153 reads. After filtering, the 16S rRNA dataset was comprised of 43 samples (Healthy n = 21, RGIS n = 22) with 10,262 OTUs and 5,571,049 reads. The OTUs were classified into 14 phyla and 216 genera.

**Fig 1:**
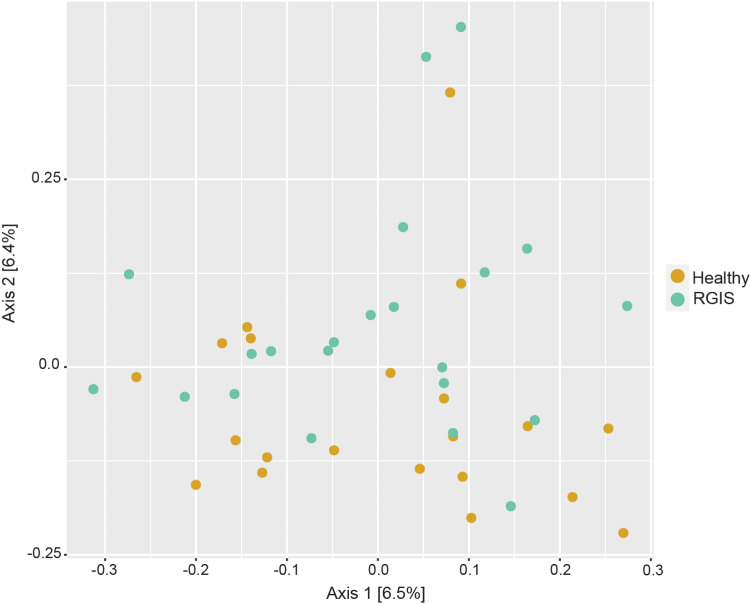
Beta diversity of bacterial microbial communities of the rabbit fecal microbiota in response to RGIS visualized as PCoA plot. Distances between samples denote Bray-Curtis dissimilarity measures based on 16S rRNA gene amplicon sequencing.

When performing whole bacterial community beta diversity comparisons, PERMANOVA results revealed significant differences (p < 0.05) in composition due to the health status of the animal. However, the unconstrained PCoA generated from the same Bray-Curtis distances did not show clustering by health status and only a relatively small amount of variation was explained by the axes (Fig 1).

Across all four metrics (number of species observed, Chao1 species richness, Shannon diversity, and Simpson evenness) tested, no significant differences (p > 0.05) in alpha diversity between healthy and RGIS rabbits were found (S1 Fig).

### Taxonomic composition of the bacterial microbiota by health status

**Fig 2:**
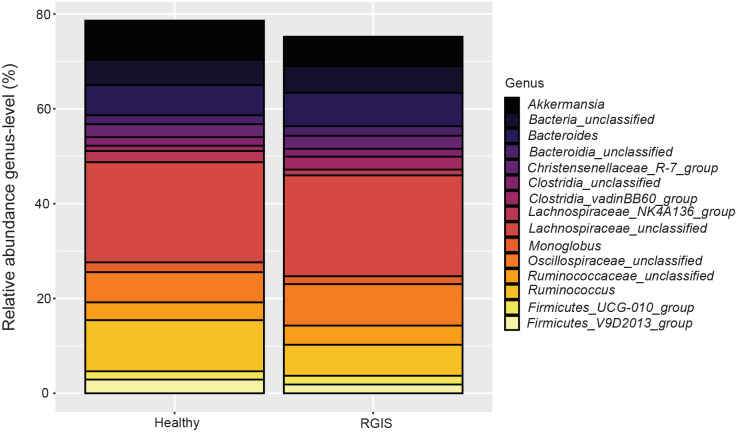
Relative abundance of the 15 most abundant bacterial genera in the feces of healthy rabbits and rabbits with RGIS.

The most abundant bacterial phyla across all groups were *Firmicutes*/*Bacillota* (Healthy 71.2%, RGIS 67.7%) *Bacteroidota* (Healthy 13.2%, RGIS 15.4%)*,* and *Verrucomicrobiota* (Healthy 8.2%, RGIS 6.1%). The most abundant genera differed based on the health status of the animal. The most abundant genera in Healthy rabbits consisted of an unclassified genus in the *Lachnospiraceae* family accounting for 21.9% of the reads, *Ruminococcus* made up 11.2%, and *Akkermansia* accounted for 8.2% of the reads (Fig 2). The three most abundant genera for RGIS rabbits consisted of an unclassified genus in the *Lachnospiraceae* family (21.1%) an unclassified genus in the *Oscillospiraceae* family (8.6%) and *Bacteroides* (7.1%). The classifications for the 15 most abundant genera for each group can be found in S1 Table. When considering the 100 most abundant genera, the relative abundance of seven bacterial genera was significantly different (p < 0.05, q < 0.05) between the Healthy and RGIS rabbits. Five genera were found in higher abundance in Healthy rabbits including: the *Lachnospiraceae*_NK4A136 group (p = 0.001, q = 0.02), the V9D2013 group (p = 0.002, q = 0.03) and the RF39 group (p < 0.001, q = 0.01) in the *Firmicutes/Bacillota* phylum, an unclassified genus in the *Erysipelatoclostridiaceae* family (p < 0.001, q = 0.01), and an unclassified genus in the *Gastranaerophilales* order (p < 0.001, q = 0.02). An unclassified genus in the *Enterobacteriaceae* family (p = 0.003, q = 0.04) and *Clostridium sensu stricto* 1 (p < 0.001, q = 0.02) were found in higher abundance in rabbits with RGIS. The effect size estimates of these changes (Log_2_ fold-change) in abundance are visualized in S2 Fig. On an OTU level, the most abundant OTUs in Healthy rabbits were OTU1 *Akkermansia* (5.5%), OTU3 *Ruminococcus* (4.1%), and OTU5 an unclassified OTU in the *Lachnospiraceae* family (3.5%). In rabbits with RGIS, the most abundant OTUs were OTU1 *Akkermansia* (3.6%), OTU2 an unclassified OTU in the *Oscillospiraceae* family (3.4%), and OTU12 an unclassified OTU in the *Lachnospiraceae* family (2.4%). OTU75 *Lachnospiraceae*_NK4A136 group was significantly more abundant (p < 0.05, q < 0.05) in Healthy rabbits compared to rabbits with RGIS.

### 18S rRNA gene amplicon sequencing

After quality control, the average sequencing depth per sample for the eukaryotic rabbit fecal microbiota dataset was 107,343 with a standard deviation of 41,844 sequences. After filtering, the 18S rRNA dataset was comprised of 42 samples (Healthy n = 20, RGIS n = 22) containing 1,867 OTUs with 4,508,428 reads. The OTUs were classified into 8 phyla and 43 genera.

**Fig 3:**
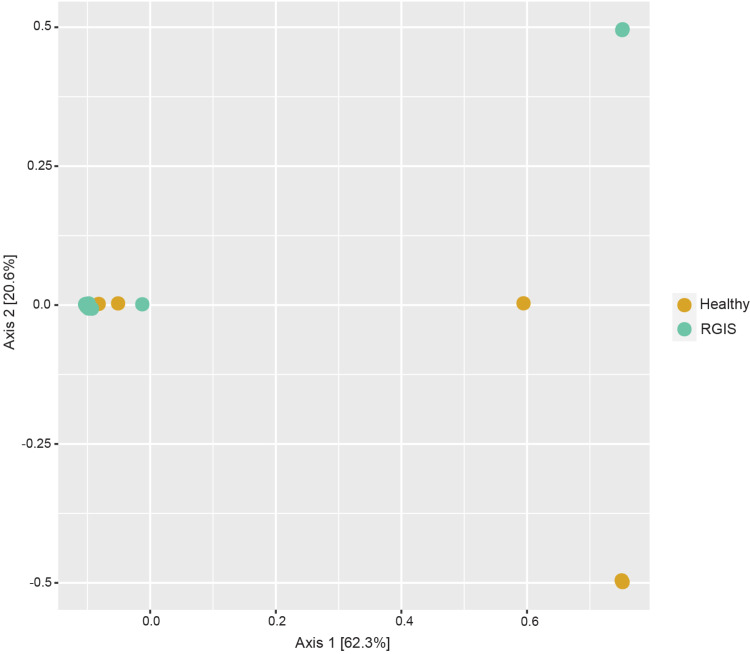
Beta diversity of eukaryotic microbial communities of the rabbit fecal microbiota in response to RGIS visualized as PCoA plot. Distances between samples denote Bray-Curtis dissimilarity measures based on 18S rRNA gene amplicon sequencing.

No significant differences in eukaryotic microbial community composition (p > 0.05) were found between healthy and RGIS rabbits using PERMANOVA. In line with this, the resulting PCoA plot revealed that most samples had very similar compositions (Fig 3).

Like the bacterial dataset, across all four metrics (Observed, Chao1, Shannon, and Simpson) tested there were no significant differences (p > 0.05) in alpha diversity based on the health status of the animal (S3 Fig).

### Taxonomic composition of the eukaryotic microbiota by health status

**Fig 4:**
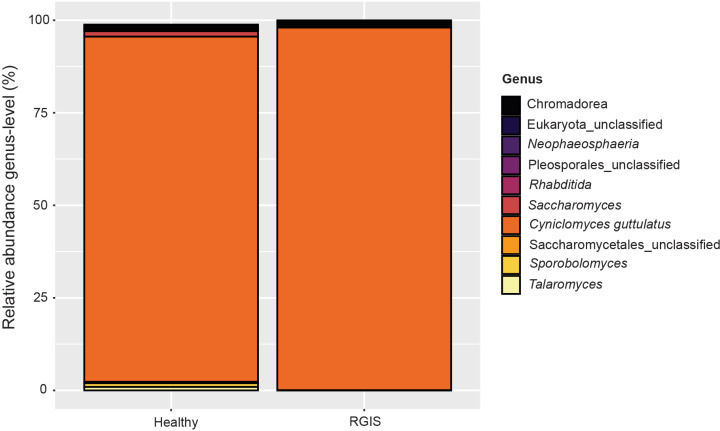
Relative abundance of rabbit GI tract eukaryotic microbial communities on genus level in response to RGIS based on 18S rRNA gene amplicon sequencing. Only the 10 most abundant genera are shown.

Ascomycota (Healthy = 99.1%, RGIS = 98.3%), Nematozoa (Healthy = 0.7%, RGIS = 1.7%), and Basidiomycota (Healthy = 0.1%, RGIS = < 0.01%) were the most abundant phyla across all sampling groups. An unclassified genus in the Saccharomycetaceae family constituted the majority of the reads in the eukaryotic dataset (Healthy = 98.7%, RGIS = 98.1%) (Fig 4). To improve classification accuracy, the consensus sequence of this OTU was classified using NCBI BLAST and was identified as *Cyniclomyces guttulatus* (99.7% identity to the type strain of *C. guttulatus,* accession no.: JQ698886.1). The most abundant genera for rabbits with RGIS differed slightly. The second and third most abundant genera included an unclassified genus in the Chromadorea (nematodes) class (1.7%) and *Saccharomyces* (0.1%). The classifications for the 15 most abundant eukaryotic genera can be found in S2 Table. When considering all 43 genera, the relative abundance of one genus was significantly different between the Healthy and RGIS rabbits. The yeast *Vishniacozyma*, was found in greater abundance (p = 0.001, q = 0.04) in Healthy rabbits. The three most abundant OTUs in Healthy rabbits were OTU1 (79.7%)*,* OTU4 (16.7%)*,* and OTU9 (0.6%) which were all identified as *Cyniclomyces guttulatus* using NCBI BLAST. The most abundant OTUs in rabbits with RGIS were OTU1 (88.1%) and OTU3 (7.7%) which were both identified as *Cyniclomyces guttulatus*, and OTU6 (1.6%) an unclassified OTU within the Chromadorea class (nematodes)*.* There was no significant difference in the relative abundance of the 100 most abundant OTUs when comparing Healthy rabbits to rabbits with RGIS.

## Discussion

Previously, anecdotal evidence led to the theory of pathogenic bacteria proliferation being included in the pathology of RGIS [2,10]. If RGIS can be associated with major changes in the GI microbiota, then it can be concluded that dysbiosis is a possible outcome of the syndrome. This study is the first study to analyze the effects of RGIS on the GI microbiota of rabbits. Here, we performed detailed characterization of the effects of RGIS on the rabbit bacterial and eukaryotic fecal microbiota. One limitation of this study is that it was not a controlled study and relied solely on client-owned animals that were admitted to participating clinics. Future research employing more standardized conditions is necessary to provide more definitive evidence for the conclusions of the present study.

### Effect of RGIS on the bacterial microbiota

On a whole community level, when considering the effect of RGIS, our study revealed a significant, albeit small, difference in the bacterial community composition (beta diversity) between healthy and RGIS rabbits. Markedly, no significant shifts in alpha diversity (species richness and evenness) were detected. This is contrary to other rabbit GI tract pathologies, such as ERE and antibiotic mediated dysbiosis, that resulted in a significant reduction in alpha diversity in GI samples [7,8,23].

The following genera were found in higher abundance in healthy rabbits: *Lachnospiraceae* NK4A136 group, the V9D2013 group in the *Firmicutes* phylum, the RF39 group in the *Firmicutes* phylum, an unclassified genus in the *Erysipelatoclostridiaceae* family, and an unclassified genus in the *Gastranaerophilales* order. As shown in S2 Fig, the difference in abundance (Healthy vs. RGIS) did not exceed a Log_2_-fold change of 2 for these genera. Similarly, previous studies found *Lachnospiraceae* NK4A136 group and V9D2013 (*Firmicutes*) group in higher abundance in healthy rabbits compared to ERE rabbits [9]. Two genera were found in higher abundance in rabbits with RGIS: *Clostridium sensu stricto 1* and an unclassified genus in the *Enterobacteriaceae* family (S2 Fig). Due to the unclassified nature of this genus, no clear conclusion about its degree of relatedness with known members of the *Enterobacteriaceae* can be made. This lack of taxonomic resolution can partially be explained by the limited taxonomic resolution of short PCR products (usually 250 to 300 bp) which are used in Illumina MiSeq amplicon sequencing. The significantly higher abundance of the genus *Clostridium* in RGIS rabbits is remarkable, as an increase of *Clostridium* has been described in rabbits with RGIS, albeit anecdotally [2,10].

Clostridial species are normally present in the GI tract of healthy rabbits and aide in the microbial degradation of plant biomass with their cellulolytic capabilities [24–27]. Furthermore, certain strains have been applied as probiotics in weaning and post-weaning rabbits [28,29]. Contrary to the otherwise beneficial role of clostridial species in the GI tract, certain *Clostridium spp*. are opportunistic pathogens linked to a variety of different systemic and enteric diseases. Examples of known rabbit pathologies associated with *Clostridium* spp. include: Tyzzer’s disease caused by *Clostridium piliforme*, necrotizing gastritis associated with *Clostridium septicum*, enterotoxemia in newly weaned rabbits is frequently associated with *Clostridium spiroforme*, and the proliferation of *Clostridium perfringens* and *Clostridium cuniculi* is known to have a principal role in ERE [7,8,30–33].

Our findings point towards the proliferation of clostridial species within the *Clostridium sensu stricto 1* cluster being associated with RGIS. Cluster 1 includes previously described opportunistic pathogens like *C. septicum* and *C. perfringens* [34]. This study is the first to survey the impact of RGIS on the bacterial microbiota and future work is needed to further characterize (using, e.g., metagenome shotgun sequencing) the proliferating *Clostridium sensu stricto 1* and *Enterobacteriaceae* bacteria. Future functional research is necessary to determine the potential role of these microorganisms in the underlying disease process. Given that the fecal samples were collected after RGIS was diagnosed, it is unclear if the proliferation of these microorganisms is a consequence or a contributing factor of RGIS.

### Effect of RGIS on the eukaryotic microbiota

This is the first 18S rRNA dataset to characterize the eukaryotic fecal microbiota of the rabbit. The eukaryotic microbiota in these rabbits is characterized by comparatively low diversity and dominated by the yeast *Cyniclomyces guttulatus*, which made up over 98% of all reads. RGIS did not significantly alter the beta or alpha diversity of the eukaryotic microbiota. Since *C. guttulatus* accounted for more than 98% of all reads, the remaining genera have considerably fewer reads. Thus, we have limited evidence to support any significant changes in relative abundance. *C. guttulatus* has long been described as a common and dominant member of the rabbit GI tract microbiota of the mucosal/epithelial surfaces of the rabbit GI tract [35,36]. Some studies have associated *C. guttulatus* with diarrhea in rabbits [37,38]; however, a direct causal link has not been established so far. Studies have found no harmful effects of *C. guttulatus* when given orally or injected intravenously in rabbits and mice, suggesting that *C. guttulatus* is not associated with disease [39–41]. Given the very high abundance of *C. guttulatus* in all samples, regardless of health status, it seems unlikely that *C. guttulatus* is associated with RGIS.

The relatively small effect size of RGIS on changes in the composition of the fecal microbiota in both the bacterial and the eukaryotic microbiota can potentially be explained by several factors. Although we took utmost care of selecting a homogeneous set of animals for this study, it is possible that the underlying differences between the animals such as: breed, handling, age, and geographic location, might blur differences in the GI tract microbiota that are due to RGIS. As this is the first study to investigate the effect of RGIS on the rabbit microbiota, the lack of data for comparison further hampers interpretation of the results. Another possible explanation for the results obtained could be that fecal samples, which were used for this study, are more representative of the hindgut than the foregut microbiota. It is well known that the microbiota of different sections of the rabbit GI tract are distinct [24,25,27]. Since reduced motility can occur in any part of the GI tract it is possible that the effects of RGIS were not accurately represented in the microbiota of fecal samples.

Future studies analyzing the microbiota of rabbits during RGIS are thus needed to increase our understanding of RGIS and links with the GI tract microbiota. Such studies could potentially include using animal trials under more controlled conditions (in contrast to sampling pet rabbits presenting with RGIS symptoms in a clinical setting as in the current study) to investigate the effect of RGIS on the GI tract microbiota in different sections of the rabbit GI tract. Also, quantitative approaches for determining the abundance of certain bacteria of interest, such as quantitative PCR, would be desirable.

## Conclusion

Overall, our study revealed a minor effect of RGIS on the eukaryotic and bacterial fecal microbiota on a whole community level, but some genera showed significantly different abundances in healthy and RGIS rabbits. Particularly the observed higher abundance of *Clostridium* in RGIS rabbits, warrants further investigation. The present study is limited by its reliance on client-owned animals, emphasizing the need for future research conducted under controlled conditions to provide more conclusive evidence on the association of the rabbit GI tract microbiota with RGIS.

## Supporting information

S1 Fig Boxplots of alpha diversity measurements of the bacterial fecal microbiota from Healthy rabbits and rabbits with RGIS based on 16S rRNA gene amplicon sequencing. The figure compares the number of observed species (Observed), species richness (Chao1), diversity (Shannon), and evenness (Simpson) based on the health status of the rabbit. There were no significant differences in alpha diversity between the groups.
(PDF)

S2 Fig Log_2_ fold-changes of genera with significant changes in relative abundance comparing rabbits with RGIS to Healthy rabbits. The white bars indicate genera that were more abundant in rabbits with RGIS. The grey bars indicate genera that were more abundant in Healthy rabbits. Error bars indicate absolute standard error of the mean.
(PDF)

S3 Fig Boxplots of alpha diversity measurements of the eukaryotic fecal microbiota from Healthy rabbits and rabbits with RGIS based on 18S rRNA gene amplicon sequencing. The figure compares the number of observed species (Observed), species richness (Chao1), diversity (Shannon), and evenness (Simpson) based on the health status of the rabbit. There were no significant differences in alpha diversity between the groups.
(PDF)

S1 Table Relative abundance of the 15 most abundant bacterial genera in Healthy rabbits and rabbits diagnosed with RGIS. (PDF)

S2 Table Relative abundance of the 15 most abundant eukaryotic genera in Healthy rabbits and rabbits diagnosed with RGIS. (PDF)
