# Supplementary material for: Effect of rabbit gastrointestinal stasis (RGIS) on the fecal microbiota of pet rabbits (Oryctolagus cuniculus)
Source: PLoS One. 2025 Feb 25;20(2):e0318810. doi: 10.1371/journal.pone.0318810 (PMC11856277; doi:10.1371/journal.pone.0318810)
Supplement: S1 Fig — The figure compares the number of observed species (Observed), species richness (Chao1), diversity (Shannon), and evenness (Simpson) based on the health status of the rabbit. There were no significant differences in alpha diversity between the groups. (PDF) [file pone.0318810.s001.pdf]

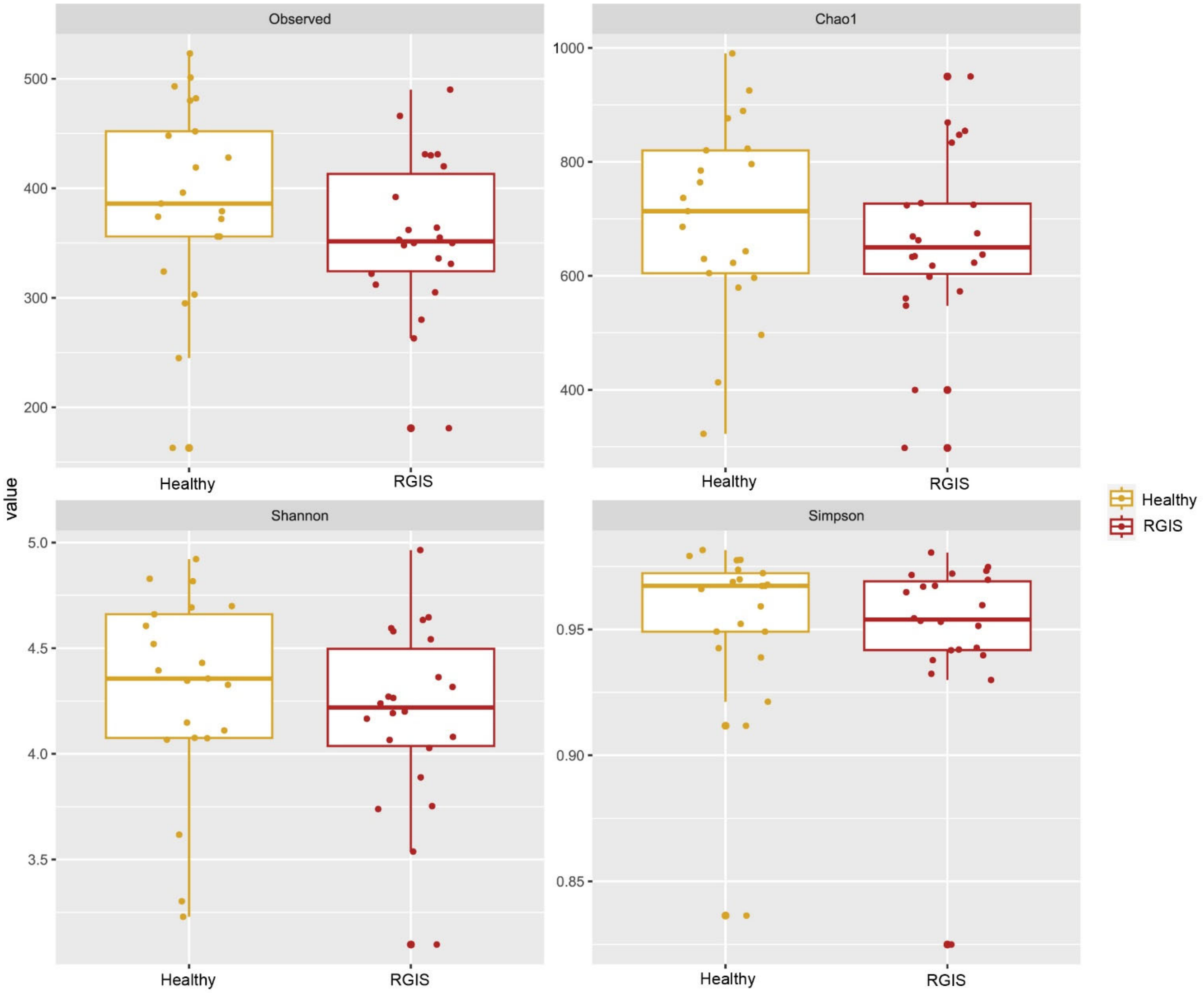

S1 Figure: Boxplots of alpha diversity measurements of the bacterial fecal microbiota from Healthy rabbits and rabbits with RGIS based on 16S rRNA gene amplicon sequencing. The figure compares the number of observed species (Observed), species richness (Chao1), diversity (Shannon), and evenness (Simpson) based on the health status of the rabbit. There were no significant differences in alpha diversity between the groups.
