# Supplementary material for: Effect of rabbit gastrointestinal stasis (RGIS) on the fecal microbiota of pet rabbits (Oryctolagus cuniculus)
Source: PLoS One. 2025 Feb 25;20(2):e0318810. doi: 10.1371/journal.pone.0318810 (PMC11856277; doi:10.1371/journal.pone.0318810)
Supplement: S2 Fig — The white bars indicate genera that were more abundant in rabbits with RGIS. The grey bars indicate genera that were more abundant in Healthy rabbits. Error bars indicate absolute standard error of the mean. (PDF) [file pone.0318810.s002.pdf]

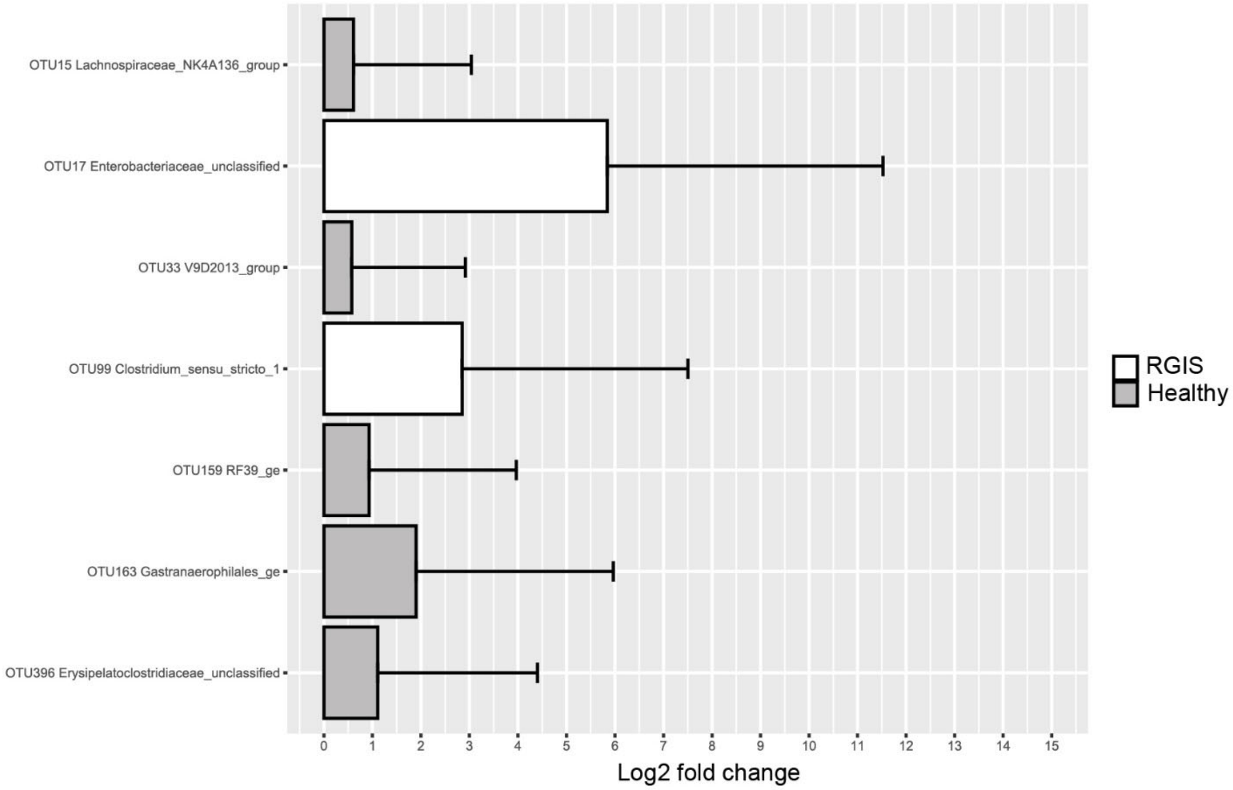

S2 Figure: Log$_2$ fold-changes of genera with significant changes in relative abundance comparing rabbits with RGIS to Healthy rabbits. The white bars indicate genera that were more abundant in rabbits with RGIS. The grey bars indicate genera that were more abundant in Healthy rabbits. Error bars indicate absolute standard error of the mean.
