# Supplementary material for: Effect of rabbit gastrointestinal stasis (RGIS) on the fecal microbiota of pet rabbits (Oryctolagus cuniculus)
Source: PLoS One. 2025 Feb 25;20(2):e0318810. doi: 10.1371/journal.pone.0318810 (PMC11856277; doi:10.1371/journal.pone.0318810)
Supplement: S1 Table — (PDF) [file pone.0318810.s004.pdf]

S1 Table: Relative abundance of the 15 most abundant bacterial genera in Healthy rabbits and rabbits diagnosed with RGIS.

Healthy		RGIS	
**(*Phylum*) *Genus***	**Relative abundance (%)**	**(*Phylum*) *Genus***	**Relative abundance (%)**
(Bacillota) Lachnospiraceae_unclassified	21.9	*(Bacillota) Lachnospiraceae_unclassified*	21.1
(Bacillota) Ruminococcus	11.2	*(Bacillota) Oscillospiraceae_unclassified*	8.6
(Verrucomicrobiota) Akkermansia	8.2	*(Bacteroidota) Bacteroides*	7.1
(Bacteroidota) Bacteroides	6.6	*(Bacillota) Ruminococcus*	6.1
(Bacillota) Oscillospiraceae_unclassified	6.0	*(Verrucomicrobiota) Akkermansia*	6.1
Bacteria_unclassified	4.7	*Bacteria_unclassified*	5.6
(Bacillota) Ruminococcaceae_unclassified	3.7	*(Bacillota) Ruminococcaceae_unclassified*	4.2
(Bacillota) V9D2013_group	3.0	*(Bacillota) Clostridia_vadinBB60_group_ge*	2.9
(Bacillota) Lachnospiraceae_NK4A136_group	2.4	*(Bacillota) Christensenellaceae_R-7_group*	2.8
(Bacillota) Clostridia_UCG-014_ge	2.3	*(Pseudomonadota) Enterobacteriaceae_unclassified*	2.2
(Bacillota) Christensenellaceae_R-7_group	2.3	*(Bacteroidota) Bacteroidia_unclassified*	2.0
(Bacillota) Monoglobus	1.9	*(Bacillota) V9D2013_group*	1.9
(Bacteroidota) Bacteroidia_unclassified	1.8	*(Bacillota) Ruminococcaceae_ge*	1.9
(Bacillota) UCG-010_ge	1.8	*(Bacillota) UCG-010_ge*	1.9
(Bacillota) Clostridia_unclassified	1.6	*(Bacteroidota) Bacteroidales_unclassified*	1.7
