# Supplementary material for: Effect of rabbit gastrointestinal stasis (RGIS) on the fecal microbiota of pet rabbits (Oryctolagus cuniculus)
Source: PLoS One. 2025 Feb 25;20(2):e0318810. doi: 10.1371/journal.pone.0318810 (PMC11856277; doi:10.1371/journal.pone.0318810)
Supplement: S2 Table — (PDF) [file pone.0318810.s005.pdf]

S2 Table: Relative abundance of the 15 most abundant eukaryotic genera in Healthy rabbits and rabbits diagnosed with RGIS

**Healthy**		**RGIS**	
**(Phylum) *Genus***	**Relative abundance (%)**	**(Phylum) *Genus***	**Relative abundance (%)**
(Ascomycota) *Saccharomycetaceae_unclassified* NCBI: *Cyniclomyces guttulatus*	98.60	(Ascomycota) *Saccharomycetaceae_unclassified* NCBI: *Cyniclomyces guttulatus*	98.11
(Nematoda) Rhabditida_ge	0.44	(Nematoda) Chromadorea_ge	1.66
(Nematoda) Chromadorea_ge	0.28	(Ascomycota) *Saccharomyces*	0.12
(Ascomycota) *Saccharomyces*	0.24	Eukaryota_unclassified	0.06
Eukaryota_unclassified	0.11	(Ascomycota) *Talaromyces*	0.02
(Ascomycota) *Talaromyces*	0.07	(Ascomycota) *Cladosporium*	0.01
(Basidiomycota) *Sporobolomyces*	0.06	(Ascomycota) *Neophaeosphaeria*	< 0.01
(Ascomycota) Saccharomycetales_unclassified	0.03	(Ascomycota) Saccharomycetales_unclassified	< 0.01
(Ascomycota) *Neophaeosphaeria*	0.02	(Basidiomycota) *Malassezia*	< 0.01
(Basidiomycota) *Vishniacozyma*	0.02	(Ascomycota) Pleosporales_unclassified	< 0.01
(Ascomycota) Pleosporales_unclassified	0.02	(Ascomycota) *Hyphozyma*	< 0.01
(Ascomycota) *Cladosporium*	0.01	(Ascomycota) *Aspergillus*	< 0.01
(Basidiomycota) *Malassezia*	0.01	(Ascomycota) *Candida*	< 0.01
(Ascomycota) *Penicillium*	0.01	(Ascomycota) *Penicillium*	< 0.01
(Ascomycota) *Aspergillus*	0.01	(Basidiomycota) Agaricomycetes_unclassified	< 0.01
